# Augmented reality and 3D visualization integrated with magnetic-assisted robotic procedures: first clinical cases using combined cutting-edge technologies

**DOI:** 10.1007/s00464-026-12841-2

**Published:** 2026-05-11

**Authors:** Mélissa V. Wills, Julio Jimenez, Marcelo Yañez, Natasha Paravic, Alejandra Seguel, Carolina Carmona, Isidora Mendez, Nicolas Balmaceda, Jerry Dang, Salvador Navarrete, Matthew Kroh, Andrew T. Strong

**Affiliations:** 1 Cleveland Clinic Lerner College of Medicine of Case Western University, Cleveland, OH USA; 2 Facultad de Medicina, Universidad de Chile, Santiago, Chile

**Keywords:** Magnetic-assisted robotic surgery, Augmented reality, 3D visualization, Minimally invasive surgery, Reduced-port laparoscopy, Surgical innovation

## Abstract

**Background:**

Current minimally invasive surgery techniques face persistent challenges: limited depth perception, assistant-dependent retraction, and added invasiveness from multiple ports. We present the first clinical experience combining three novel technologies: Magnetic-Assisted Robotic Surgery (MARS), 3D visualization, and augmented reality (AR).

**Methods:**

This IRB-approved retrospective review included 10 patients who underwent reduced-port laparoscopic surgery using the MARS system with Meta Quest 3 AR headset and EinsteinVision 3D camera in October 2024. Procedures included cholecystectomy (*n* = 4), oophorectomies (*n* = 2), Roux-en-Y gastric bypass (*n* = 2), combined cholecystectomy with sleeve gastrectomy (*n* = 1), and hiatal hernia repair (*n* = 1).

**Results:**

Median operative time was 108 min (range: 44–218 min). Each procedure required at least one fewer port than traditional approaches. No complications occurred, and there were no readmissions or protocol deviations within 30 days. Surgeons reported greatly improved visualization compared with standard laparoscopy, and no device malfunctions were encountered.

**Conclusion:**

Integrating MARS, 3D visualization, and AR proved feasible and safe across multiple surgical specialties. This platform enhanced visualization and reduced invasiveness while maintaining efficiency and safety.

Minimally invasive surgery (MIS) has evolved rapidly from open procedures to laparoscopic and robotic approaches, significantly improving patient outcomes through reduced pain, blood loss, and recovery time. Despite these advances, current MIS techniques face certain challenges. The traditional requirement for multiple ports to achieve triangulation and retraction increases invasiveness [1]. Cholecystectomy, the most performed laparoscopic procedure in the U.S. and worldwide, continues to be carried out using the traditional four-port technique. This technique, developed more than 30 years ago, has not reduced the number of incisions, even with technological advances such as console-based robotic platforms [2].

In the visualization front, two-dimensional imaging systems limit depth perception, creating steep learning curves for surgeons. And while three-dimensional imaging is available on laparoscopic platforms, instability of the handheld camera can limit visibility and its more widespread adoption. Effective procedures often require skilled assistants whose level of experience directly impacts surgical safety. These constraints remain a persistent barrier to minimally invasive approaches across surgical specialties, may not be mitigated by current robotic platforms, and highlight the need for new technological solutions [3–5].

Several emerging technologies show promise in addressing the limitations of conventional MIS approaches. Three-dimensional (3D) visualization systems have been shown to enhance depth perception while shortening learning curves for surgeons. Itani et al*.* demonstrated that 3D visualization reduced operative time by 13.5% in laparoscopic total gastrectomy and 21.8% in laparoscopic distal gastrectomy compared to conventional 2D systems, primarily by reducing intra-corporeal dissection time [6]. Surgeons also demonstrated greater technical confidence when using 3D systems, preferring more advanced techniques like intra-corporeal knot tying. Similarly, Kunert et al. showed that 3D visualization significantly shortened the learning curve, enabling faster skill acquisition with greater precision [7, 8].

Augmented reality (AR) technology represents a novel advancement in surgical visualization, utilizing head-mounted displays that position a screen millimeters from the surgeon’s eyes to deliver scalable operative field views. This offers real-time guidance and anatomical overlays without line-of-sight constraints, and has been successfully implemented across multiple specialties, including existing robotic platforms. Previous studies have demonstrated a significant impact of AR on surgical performance and cognitive load. Liao et al*.* demonstrated that AR displays reduced procedure time during ultrasound-guided interventions by unifying the visualization of patient anatomy and imaging data, thereby eliminating disruptive visual transitions between screens and the procedural field [9]. Similarly, a systematic review by Xiong et al*.* found that AR significantly improved laparoscopic training outcomes, enhanced technical skills scores, and reduced cognitive burden [10]. By integrating important information into the surgeon’s field of view, AR enhances spatial awareness and procedural efficiency in the operating room.

The Magnetic-Assisted Robotic Surgery (MARS) system from Levita Magnetics employs magnetic surgical retraction to reduce invasiveness and dependence on the assistant. This FDA-approved platform uses deployable magnetic tissue graspers manipulated with an external magnet, eliminating the need for additional ports [11]. Spurzem et al. demonstrated the feasibility of a reduced-port technique in ambulatory cholecystectomy. Steinberg et al. demonstrated that magnetic retraction reduces the need for a fourth robotic arm while maintaining tissue exposure during prostatectomy, and Larenas et al*.* showed that MARS enables independent operation with enhanced visualization and faster skill acquisition, creating a unified platform that addresses visualization challenges while reducing port requirements [12–17].

The integration of these three technologies (the MARS system, virtual/augmented reality headset, and 3D camera with custom AR application) creates a unified platform that addresses multiple visualization and technical challenges in minimally invasive surgery. This combination brings together magnetic retraction, 3D imaging, and augmented reality within a single surgical system [13, 18, 19].

Despite advances in magnetic retraction, 3D visualization, and augmented reality technologies individually, a significant knowledge gap exists regarding their combined clinical implementation. The present study addresses this gap by evaluating the feasibility, safety, and effectiveness of integrating these three complementary technologies across multiple surgical specialties. By testing this novel platform in various procedure types, we assess its generalizability and potential applications throughout surgical practice. This represents the first clinical combination of the MARS robotic platform, 3D visualization, and AR technology in minimally invasive surgery.

## Methods

### Study design

This IRB-approved retrospective review analyzed electronic medical records from patients who underwent reduced-port laparoscopic surgery assisted by the MARS system with integrated 3D visualization and augmented reality in October 2024. The study was designed to evaluate the feasibility, safety, and early clinical outcomes of this novel integration.

### Surgical team

Procedures were performed by four experienced laparoscopic surgeons: three general surgeons specializing in bariatric and minimally invasive surgery (J.J., 12 years of experience; M.Y., 6 years; N.P., 6 years) who performed the bariatric and cholecystectomy cases, and one gynecologic laparoscopist specializing in endometriosis (A.S., 10 years) who performed both oophorectomies. Each surgeon performs more than 200 laparoscopic cases annually.

### Technical setup

**Fig. 1:**
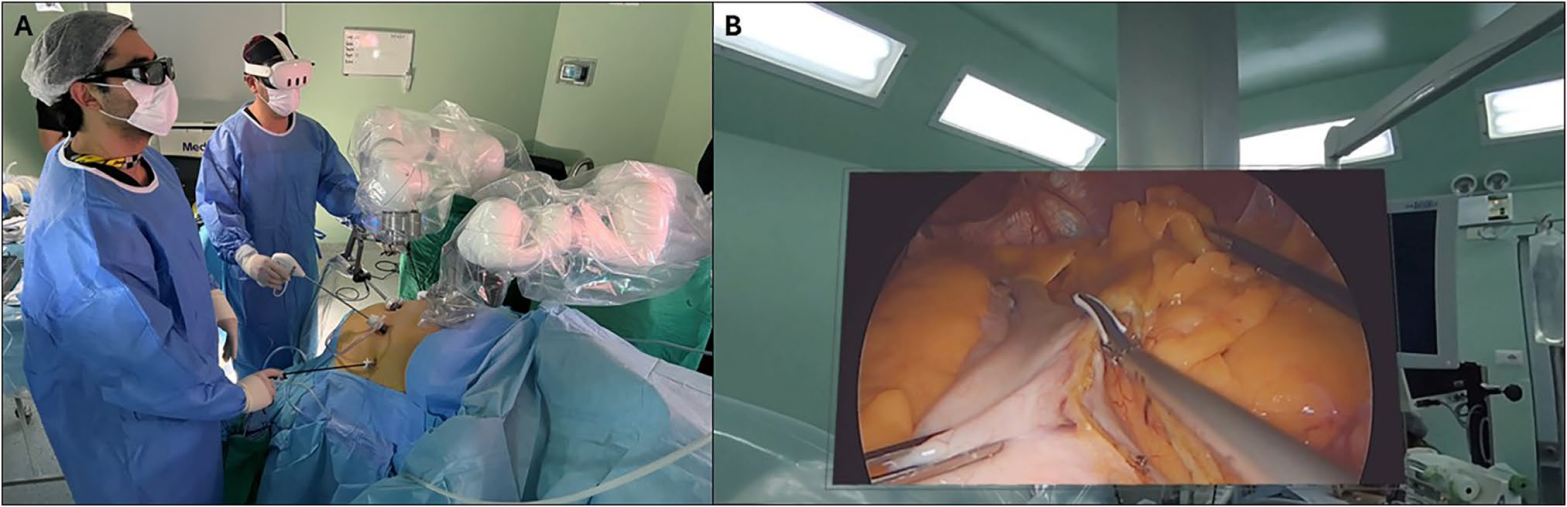
Clinical implementation of integrated MARS-AR-3D system. **A** Intraoperative view of laparoscopic sleeve gastrectomy utilizing the integrated MARS magnetic retraction system with AR headsets and 3D visualization technology. **B** Surgeon’s unobstructed view through the AR headset displaying enhanced 3D laparoscopic imaging without external monitor dependency

The technical setup consisted of these integrated components (Fig. 1):

1. MARS System: The Magnetic-Assisted Robotic Surgery system (Levita Magnetics, Mountain View, California) is an FDA-approved robotic platform that utilizes magnetic surgical retraction to reduce invasiveness while improving visualization during laparoscopic procedures. The magnetic grasper retracts one structure at a time and can be repositioned throughout the procedure to retract the liver, gallbladder, or other intraperitoneal structures as needed.
2. Meta Quest 3 Headset: An AR headset that provides an advanced digital and dynamic screen while enabling accurate spatial awareness and preservation of operative situational awareness for the surgeon in the surgical field.
3. B. Braun/Aesculap EinsteinVision 3D Camera: A three-dimensional visualization system that provides enhanced depth perception during minimally invasive procedures.
4. Custom AR Application: Levita Magnetics developed a proprietary AR application that integrates the MARS system with the Meta Quest 3 headset and the EinsteinVision 3D camera. This application was released as a commercial add-on for the MARS system for use outside the United States

### Statistical analysis

Statistical analyses were conducted using Python version 3.11.5. Continuous data were expressed as means and standard deviations for normally distributed data or as medians and ranges for non-normally distributed data. Categorical variables were reported as frequencies and percentages.

## Results

### Patient demographics

A total of 10 patients were included in this study, consisting of 9 females (90%) and 1 male (10%). The mean age in the cohort was 50.4 ± 12.7 years, with ages ranging from 32 to 72 years across the different procedure types (Table 1).

**Table 1:** Demographic characteristics of patients by laparoscopic surgical procedure type

Variable	Cholecystectomy	Oophorectomy	Roux-en-Y gastric bypass	Combined cholecystectomy and sleeve gastrectomy	Hiatal hernia repair
Number of procedures	4 (40%)	2 (20%)	2 (20%)	1	1
Age (years)	43.5 (34–52)	54 (44–64)	52 (49–55)	32	72
Sex (*N*, %)					
Female	4 (100)	2 (100)	2 (100)	1 (100)	0 (0)
Male	0 (0)	0 (0)	0 (0)	0 (0)	1 (100)

Age in years is represented as median (range)

### Surgical case distribution

**Fig. 2:**
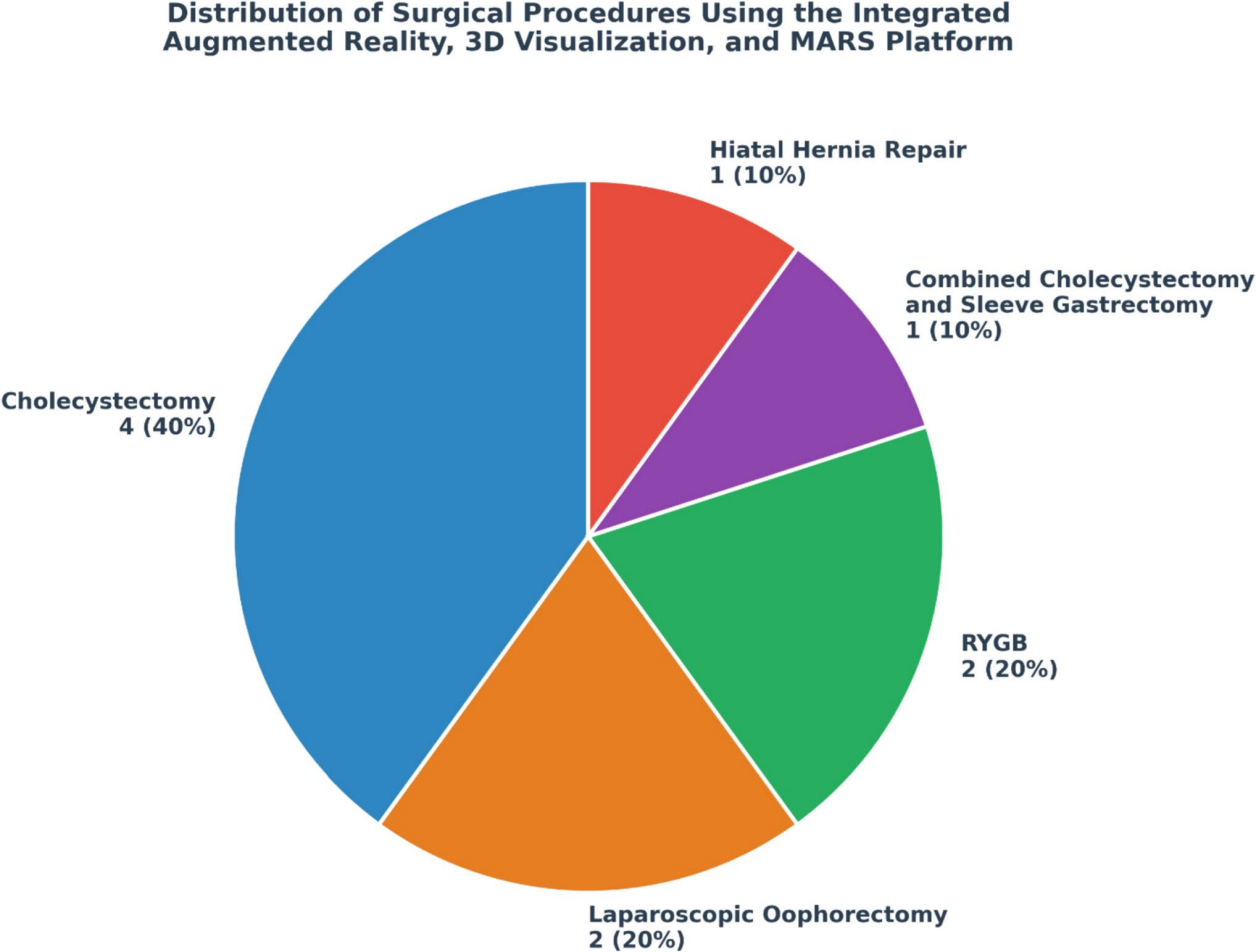
Distribution of surgical procedures using the integrated augmented reality, 3D visualization, and MARS platform

The procedures performed included four laparoscopic cholecystectomies (40%), two laparoscopic oophorectomies (20%), two laparoscopic Roux-en-Y gastric bypasses (20%), one combined laparoscopic cholecystectomy with laparoscopic sleeve gastrectomy (10%), and one giant hiatal hernia repair (10%) (Fig. 2).

### Operative outcomes

The median operative time across all procedures was 108 min (range: 44–218 min). Laparoscopic cholecystectomies (*N* = 4) had a median operative time of 72.25 min (range: 57–97 min), while the oophorectomies (*N* = 2) required 74.5 min (range: 44–105 min). Laparoscopic Roux-en-Y gastric bypasses (RYGB) (*N* = 2) had a median duration of 189 min (range: 160–218 min). The combined laparoscopic cholecystectomy with sleeve gastrectomy (*N* = 1) required 95 min, and the hiatal hernia repair (*N* = 1) required 170 min. None of the 10 surgeries resulted in any 30-day postoperative complications. The complication rate was 0% across all procedure types. Similarly, there were no hospital readmissions recorded within the 30-day postoperative period. All procedures proceeded according to hospital protocols, with no reported deviations from standard care pathways (Table 2).

**Table 2:** Operative time and postoperative outcomes by surgical procedure

Variable	Cholecystectomy	Oophorectomy	Roux-en-Y gastric bypass	Combined cholecystectomy and sleeve gastrectomy	Hiatal hernia repair
Operative time (minutes)	72.25 (57–97)	74.5 (44–105)	189 (160–218)	95	170
30-day post-operative complications (*N*, %)	0	0	0	0	0
30-day readmission (*N*, %)	0	0	0	0	0
Deviation from hospital protocol (*N*, %)	0	0	0	0	0

Operative time in minutes is represented as median (range)

### Surgical technique and technical performance

The implementation of the MARS system with magnetic retraction reduced the number of incisions by at least one across all procedures compared to traditional laparoscopic methods. Specifically, all procedures were performed using 3 ports rather than the conventional 4, with the magnetic grasper replacing the epigastric port for gallbladder and liver retraction. The cholecystectomies and gynecological procedures were performed with the surgeon without any other assistant. The integration of the MARS robotic system with the Meta Quest 3 headset and B. Braun/Aesculap EinsteinVision 3D camera was successfully executed in all cases with no device malfunctions. All surgeons reported "greatly improved" visualization compared to standard laparoscopic approaches. The elimination of polarized glasses and external monitors through the AR headset delivery system was noted to enhance the surgical experience, as well as eliminate the need for one of the two laparoscopic monitors traditionally utilized in these cases.

## Discussion

This study presents for the first time clinical integration of three cutting-edge technologies in minimally invasive surgery: the MARS robotic system, 3D visualization, and Augmented Reality (AR). Our findings demonstrate successful implementation across multiple surgical specialties without complications. The MARS system enabled the reduction of at least one port in each procedure, enhancing minimally invasive approaches while maintaining safety. Using AR and the 3D camera, surgeons consistently reported "greatly improved" visualization compared with standard laparoscopic techniques.

The combined advantages of these technologies were demonstrated through enhanced depth perception from the 3D-AR combination, simplified technical demands via magnetic retraction, and improved workflow efficiency. Despite simultaneously introducing three different technologies, operative times remained comparable to conventional approaches, suggesting that the learning curve did not significantly impact procedural duration. These findings indicate that the thoughtful integration of complementary surgical technologies can enhance surgical capabilities without compromising safety or efficiency.

The findings in the present study align with other emerging evidence on new technological applications in minimally invasive surgery. The absence of complications in our series corroborate the findings from multiple systematic reviews, including an analysis by Doornbos et al. of 22 articles on AR in minimally invasive surgery of deformable organs and Sumdani et al. review of spine surgery applications, both of which demonstrated favorable outcomes without increased procedural complications across various surgical specialties [20, 21]. Furthermore, 3D visualization systems in surgery have been shown to improve clinical outcomes and maintain safety, with studies demonstrating significantly reduced blood loss and shorter hospital stays without increasing perioperative morbidity or complications [22–25]. No intraoperative or postoperative complications were reported in our series while utilizing this approach. It also reduces the number of laparoscopic ports required during surgery while maintaining surgical efficacy and safety standards, which may translate to decreased postoperative pain, improved outcomes, and accelerated recovery [12, 22, 26]. This consistency supports the notion that combining advanced visualization technologies with advanced surgical approaches does not compromise patient safety or increase procedural risk.

Regarding operative efficiency, our procedural durations were comparable to conventional approaches despite implementing multiple new technologies simultaneously. This aligns with findings reported by Brockmeyer et al., including that properly designed AR systems maintain procedural efficiency without extending operative times [27]. 3D visualization studies show maintained efficiency with improved depth perception and spatial awareness. For MARS technology, Steinberg et al. reported efficient mean operative times for reduced-port procedures [12], with Larenas et al. documenting significant improvements in docking times after just four cases [13]. In this context, our experience suggests that even during initial implementation phases, the integration of these technologies does not significantly extend operative times—a critical consideration for workflow integration. It also indicates that when technologies are carefully designed with surgeon-specific workflow and needs in mind, their implementation becomes more natural, minimizing the learning curve and facilitating smoother adoption.

There are several ways in which our study distinguishes itself from prior AR implementations. First, our approach eliminates the need for polarized glasses, a limitation explicitly outlined previously. These polarized systems constrain visualization capabilities by reducing light transmission, limiting peripheral vision, and creating discomfort during longer procedures [20, 27]. While our system does introduce a headset requirement, the Meta Quest 3 provides a dynamic screen while enabling spatial awareness and eliminating the need for external monitors, which may help to streamline setup in the operating room. Second, whereas previous approaches struggled to maintain tracking accuracy when surgical instruments temporarily occluded anatomical structures, our system maintained precise visualization throughout the entire procedure, including during challenging surgical maneuvers [28]. Third, the integration of magnetic retraction with AR represents a novel technical combination previously unexplored in the surgical literature, creating unique opportunities for reducing invasiveness, and improving visualization. Fourth, our direct 3D visualization embedding overcomes long-standing depth perception limitations in AR implementations, as substantiated by systematic reviews [29]. Finally, the clear workflow integration between the MARS system, Meta Quest 3 headset, and EinsteinVision 3D camera enables surgeons to navigate complex anatomical environments with unprecedented precision and clarity [30].

Beyond surgical outcomes, this integrated technology platform may offer substantial educational and workflow advantages. The immersive visualization provided by the Meta Quest 3 headset could create new opportunities for surgical training by allowing trainees to experience the procedure with enhanced 3D depth perception and AR overlays [31], even if not physically present in the operating room. Unlike conventional teaching with polarized glasses and external monitors, this system may enable better spatial resolution of complex anatomy and procedural steps early in the surgical career. Additionally, the potential to incorporate medical records and imaging data directly into the surgeon's field of view represents a significant advancement that could not only eliminate the need to consult external references during critical moments, but also prevent distracting context-switching between the surgical field and the monitors, thereby facilitating a “flow state” that is typically associated with optimal performance [32, 33]. Additionally, the platform supports live teleconsultation, enabling surgeons to broadcast their perspective to distant specialists for guidance during complicated cases—potentially even serving as a gateway toward fully remote surgery [32]. While our study demonstrates technical improvements with the AR-3D-MARS integration, future research should formally evaluate its promising potential to enhance surgical training and alleviate cognitive demands on operating surgeons. 

While not explicitly evaluated in this study, the combination of these technologies may offer additional advantages in terms of operating room efficiency. The MARS system functions as an assistant to the laparoscopic surgeon and can often replace a human operating assistant in many cases. The elimination of one laparoscopic screen from the operating room also reduces setup complexity and allows for greater flexibility in positioning the operating table and additional surgical equipment.

The cost-effectiveness of this integrated platform warrants further investigation. While formal economic analysis was not performed in this study, several factors may contribute to favorable resource utilization. Reducing the number of ports decreases disposable instrument costs per case. The capacity of the MARS system to replace a human assistant may reduce staffing requirements in high-volume centers. Additionally, potential reductions in postoperative pain and length of stay associated with reduced-port approaches could translate into measurable savings for hospitals and payers. Prospective economic analyses comparing this platform to conventional 2D laparoscopy and console-based robotic systems are needed to formally quantify these effects.

In terms of limitations to this initial study, first, our sample size of 10 patients is smaller than certain published series, though this is typical for early feasibility work. Additionally, the single-arm design, which lacks a comparative group, and the heterogeneity of the procedures performed limit the generalizability of our findings. Our study did not assess the learning curve, cost-effectiveness, or economic impact of implementing this integrated platform. The results may be influenced by the experience and skill of the participating surgeons and teams, and we did not formally evaluate the ergonomics or user experience associated with the system. Additionally, patient selection criteria were not discussed in detail, and certain patient factors could potentially influence the system’s performance or outcomes. As with the existing literature, long-term follow-up data are lacking in the present series. Larger, procedure- and specialty-specific comparative studies, including randomized controlled trials, will be essential to more comprehensively evaluate the clinical impact, safety profile, and advantages of this integrated system compared to conventional laparoscopic techniques.

## Conclusion

This study demonstrates the successful clinical integration of three complementary technologies—the MARS robotic system, 3D visualization, and augmented reality—in minimally invasive surgery across multiple specialties. Our findings indicate that this novel combined platform enhances surgical visualization, reduces invasiveness, and maintains procedural efficiency without compromising safety or outcomes. Each procedure reduced the number of ports compared with conventional approaches, and surgeons consistently reported improved visualization quality. Despite the potential complexity of integrating multiple technologies, the learning curve did not significantly impact operative times. Our initial results indicate that integrating magnetic retraction, 3D imaging, and AR shows significant potential for advancing minimally invasive surgery.
